# Fast and accurate methods for phylogenomic analyses

**DOI:** 10.1186/1471-2105-12-S9-S4

**Published:** 2011-10-05

**Authors:** Jimmy Yang, Tandy Warnow

**Affiliations:** 1 Department of Computer Science, University of Texas at Austin, Austin TX 78712

## Abstract

**Background:**

Species phylogenies are not estimated directly, but rather through phylogenetic analyses of different gene datasets. However, true gene trees can differ from the true species tree (and hence from one another) due to biological processes such as horizontal gene transfer, incomplete lineage sorting, and gene duplication and loss, so that no single gene tree is a reliable estimate of the species tree. Several methods have been developed to estimate species trees from estimated gene trees, differing according to the specific algorithmic technique used and the biological model used to explain differences between species and gene trees. Relatively little is known about the relative performance of these methods.

**Results:**

We report on a study evaluating several different methods for estimating species trees from sequence datasets, simulating sequence evolution under a complex model including indels (insertions and deletions), substitutions, and incomplete lineage sorting. The most important finding of our study is that some fast and simple methods are nearly as accurate as the most accurate methods, which employ sophisticated statistical methods and are computationally quite intensive. We also observe that methods that explicitly consider errors in the estimated gene trees produce more accurate trees than methods that assume the estimated gene trees are correct.

**Conclusions:**

Our study shows that highly accurate estimations of species trees are achievable, even when gene trees differ from each other and from the species tree, and that these estimations can be obtained using fairly simple and computationally tractable methods.

## Background

With the increased availability of whole genome sequence assemblies, the estimation of species trees based upon the entire genome is now possible. The most frequently used approaches for estimating species phylogenies compute alignments on each gene, concatenate these alignments into one super-alignment, and then estimate a tree from the super-alignment. However, these “combined analysis” methods do not have good statistical properties because different regions of the genome can have different evolutionary histories. More generally, it is increasingly clear that gene trees can be different from species trees due to a number of biological processes. One of the dominant causes for this incongruence between gene and species trees is *incomplete lineage sorting* (ILS) [1], a population-level process where lineages “coalesce” [2] deeply in the species phylogeny, so that the gene tree can be different from the species tree. Thus, coalescence is a “backwards-in-time” process, which is mathematically equivalent to a forwards-in-time process called “lineage sorting”, whereby alleles within an ancient population diverge before a speciation event, and then assort into the two sub-species. ILS has been implicated in the different hypotheses for the evolutionary tree on human, chimp, and gorilla [3], but ILS is quite generally a major problem for species tree estimation [4].

Several methods have been developed that estimate species trees from estimated gene trees under the ILS model. One main approach is to find the species tree that implies the fewest number of deep coalescent events, a computational problem called “MDC” (for minimize deep coalescences [1]), a problem that is NP-hard when the gene trees can be on different taxon sets [5], but of unknown computational complexity when the gene trees are constrained to be on the same taxon set. Heuristics for MDC have been implemented in various packages, including Phylonet [6], iGTP [7], and Mesquite [8]. Optimal solutions to MDC are not guaranteed to be statistically consistent, meaning that even with true trees for an unboundedly large number of genes, the species tree optimizing the MDC criterion may not converge to the true tree [9]. Similarly, standard consensus tree methods have been shown to not be statistically consistent [10] (however, see [11,12]).

Alternative techniques that are based upon statistical models for ILS include GLASS [13], STEM [14], *BEAST [15], BEST [16], and BUCKy [17,18] (see the review article of Degnan and Rosenberg [19] for more on these methods). GLASS is a polynomial time distance-based method (available in the Phylonet package [6]) that is statistically consistent when all genes evolve under the same rate; thus, when given a sufficient number of correct gene trees, GLASS will return a correct species tree with high probability. STEM is a statistical method that requires that all input gene trees be properly rooted, and also has statistical guarantees [14] when the gene trees are correct. *BEAST, BEST, and BUCKy [17] are Bayesian methods that produce a distribution of species tree topologies based upon ILS from gene sequence alignments; BUCKy also explicitly considers causes of discord other than ILS (e.g., horizontal gene transfer).

Other new methods have been developed and studied for estimating species trees from incongruent gene trees, among them the method by Yu et al. [20] (and available in Phylonet) for a variant of the MDC approach, which we call “constraint-MDC”. The input to constraint-MDC is a set of estimated gene trees, where the trees can be unrooted and only partially resolved, and the objective is to find a species tree and rooted binary refinements of these estimated gene trees so as to optimize the MDC criterion with respect to the rooted binary refinements of the estimated gene trees. Thus, each estimated gene tree topology is considered to be a constraint on the topology of the true gene tree.

Simulation studies to evaluate these methods have examined performance on datasets in which the gene trees can differ from the species tree due to ILS. These studies have shown that the methods in Phylonet for Constraint-MDC produce highly accurate species trees [20], and that statistical species tree estimation (BEST, *BEAST, BUCKy, and Stem) can produce more accurate tree than the combined analysis method that computes trees directly from the concatenated sequence alignment [15]. They have also shown that *BEAST produces more accurate trees than BEST [15], that BEST and BUCKy produce more accurate trees than Stem [21], and that Stem produces more accurate trees than MDC-based analyses [22]. However, all these studies are substantially limited because of the restriction to small datasets (with at most 17 taxa), the use of only substitutions (i.e., no indels) in the sequence evolution models, and the limitation to ILS for causes of incongruence. A recent study [23] addressed this last limitation, and compared BEST and BUCKy on datasets in which the gene trees could differ from the species tree due to HGT (horizontal gene transfer) as well as ILS, and found cases where BUCKy gave more accurate reconstructions.

However, the other restrictions are still too limiting, for the following reasons. First, the restriction to small datasets does not allow us to evaluate relative performance on larger datasets, and does not help us understand the computational limitations of the methods, some of which are quite intensive. The restriction to substitution-only sequence evolution models means that alignments do not need to be estimated, and this reduces errors in estimated gene trees (especially for large datasets [24]). The two conditions together make accurate gene tree estimation easier than for the general case, and especially easier than for larger datasets with high rates of indels and substitutions for which accurate alignment estimation is particularly challenging.

In this paper we report on a simulation study using ROSE [25] in which we explore the performance of a large number of species tree estimation methods on nucleotide sequence datasets with 17 to 500 taxa that evolve with substitutions as well as indels. We report results for two experiments. The first experiment explored performance on datasets with multiple genes on 17 or 100 taxa in which the true gene trees can differ from the true species tree (and hence from each other). In the second experiment, we explored performance on datasets with multiple genes on 100 and 500 taxa in which the true gene trees are topologically identical but can have different branch lengths. Thus, the first experiment evaluates performance when the estimated gene trees can differ due to ILS as well as estimation error, while the second experiment focuses on performance when all incongruence between estimated gene trees is due to estimation error.

Our simulation protocol is substantially more complex (and more realistic) than most prior studies. Except for the 17-taxon datasets (the same ones used in [20], and which evolve under the Jukes-Cantor [26] model, a simple substitution-only model in which all substitutions are equally likely), all our datasets evolve under a model that includes indels (insertions and deletions) as well as substitutions. We use a more general substitution model, GTR+Gamma (the General Time Reversible model) [27], using GTR+Gamma parameters estimated for a biological dataset (see [28] for details for this simulation model), instead of Jukes-Cantor. We examine 100- and 500-taxon datasets, thus greatly exceeding the maximum taxon dataset size (17) used in prior studies.

For datasets that evolve with indels, we estimate alignments using the default setting for MAFFT v. 6.717b [29], a multiple sequence alignment method that is established to be among the most accurate [24,28]. We estimate gene trees using the default setting for FastTree [30] v. 2.1.3 to produce a fast and approximate solution to maximum likelihood under GTR+Gamma. We estimate distributions of gene trees using the default setting for RAxML [31] v. 7.2.6 with 100 rapid bootstrap replicates and a Bayesian method (MrBayes [32] v. 3.1.2). The RAxML analysis includes a “best” ML tree resulting from its search, as well as a set of gene trees, allowing us to produce bootstrap support values for the edges of its best tree. For our default MrBayes analysis, we ran MrBayes for 1,000,000 generations with a burn-in of 25% under the GTR+Gamma+I model; all other options were left as defaults. With two runs and a sampling ratio of 1:100, this is designed to produce 15,000 trees per gene sequence alignment input. We varied the number of MCMC iterations used within MrBayes analyses, and we also varied the number of the sampled trees from the MrBayes analyses used in the BUCKy analyses.

Although MrBayes and RAxML with rapid bootstrapping produce a sample of different trees, we also use them to produce point estimates of the gene tree for those species tree estimation methods that require single trees for each gene. In the case of the MrBayes (MB) analysis, we output the majority consensus tree (MB-maj) (that is, the tree containing all the splits that appear in more than half of the trees that are sampled), and the maximum a *posteriori* probability (MB-map) tree. For the RAxML analysis, we use RAxML-75%, the 75%-bootstrap tree (i.e., the best ML tree found by RAxML during the bootstrap search, with all branches with support below 75% contracted). Finally, FastTree outputs support values for every branch in the trees it produces; thus, we can also use FT-75%, the tree produced by contracting all branches with support less than 75% in the FastTree output. Note that the MP-maj, RAxML-75%, and FT-75% trees are not likely to be fully resolved; therefore, these point estimations of gene trees can only be used with species tree estimation methods that permit unresolved gene tree inputs.

We explore seven methods for estimating species trees from estimated gene trees or gene tree distributions. Four of these are based upon ILS, and include Glass (from Phylonet v. 2.3), Phylonet-MDC v. 2.3 (here called “Phylonet”), iGTP-MDC v. 1.1, and BUCKy v. 1.4.0. The other three methods are Greedy, iGTP-Dup v. 1.1 and iGTP-Duploss v. 1.1. All of these methods, except BUCKy, take as input a single tree for each gene. In contrast, BUCKy operates in two steps: first it uses a technique (the default is MrBayes) to produce a distribution of estimated gene trees for each gene, and then it uses these distributions to infer the species tree. In fact, though, BUCKy produces two different trees–the “concordance” tree (which we refer to as “BUCKy-con”) and the population tree (which we refer to as “BUCKy-pop”) [18]. The key difference between these two outputs is that the population tree is based upon a statistical model for incomplete lineage sorting while the concordance tree is not, and thus the concordance tree is designed to be used under more general conditions than ILS. The three iGTP methods (iGTP-mdc, iGTP-dup, and iGTP-duploss) each uses the same basic algorithmic search strategy within iGTP but seek to optimize different criteria (the number of deep coalescences, number of duplications, or number of duplications and losses, respectively). Finally, “Greedy” is the greedy consensus technique (also called the “extended majority” consensus) using PAUP* [33] v. 4.0b10; it begins by computing the majority consensus (the tree whose edge-induced taxon bipartitions are those that appear in more than half of the input trees), and then adds bipartitions (if compatible) to the consensus tree, in an order reflecting the frequency with which each bipartition appears. Note that Phylonet and Greedy can be applied to incompletely resolved gene trees, and that when we use Phylonet on unresolved gene trees, it attempts to find optimal solutions to the Constraint-MDC problem. As discussed earlier, we produce incompletely resolved gene tree estimations either by contracting low support branches in fully resolved estimated gene trees, or by computing the majority consensus tree of the distribution produced by MrBayes.

We describe methods for species tree estimation by indicating the technique used to estimate the gene trees (or their distributions) and the technique used to compute the species tree from the gene trees. For example, Greedy(FT-75%) refers to the method that computes the FT-75% tree on every gene sequence alignment, and then combines these (potentially unresolved) trees using Greedy. Similarly, BUCKy-pop(MB-spa) refers to BUCKy-pop run on a sparse sample of the MrBayes distributions for each gene, BUCKy-con(MB-full) refers to BUCKy-con run on the full sample of the MrBayes distributions, and Phylonet(MB-map) refers to Phylonet run on the MAP trees from the MrBayes distributions. Not all species trees we compute are fully resolved; hence, the Robinson-Foulds distance (i.e., the bipartition distance), is inappropriate for evaluating accuracy, as it is biased in favor of unresolved trees [34]. Therefore, we report tree error using the missing branch rate, which is the proportion of internal branches in the true tree defining bipartitions that are missing in the estimated tree, also known as the “false negative” (FN) rate. However, many of the estimated species trees are fully resolved, and for these trees, this measure is identical to the Robinson-Foulds metric. These error rates range from 0.0 - 1.0, with error of 0.0 indicating that the estimated tree is identical to the true tree.

## Results

### Computational issues

We began by exploring the computational performance of the methods we studied on 12 100-taxon datasets, half with 25 genes and half with 50 genes. These studies showed that these different pipelines (estimate gene trees or distributions on gene trees, then use these to estimate the species tree) vary substantially in terms of computational effort.

The most computationally intensive methods we explored use MrBayes or RAxML to estimate distributions on gene trees, but MrBayes analyses are particularly expensive. Our analyses (data not shown) indicated that enforcing a requirement that all MrBayes analyses reach convergence was not achieved on any dataset we examined, even after at least a week for each alignment (and more than two weeks for some alignments). Therefore, each 17-taxon 32-gene dataset we examined could easily require a year (perhaps several years) of analysis in order for each MrBayes analysis to reach convergence.

Restricting the MrBayes runs so that each gene is analyzed in at most a day greatly reduces the running time, and allows the first step (computing gene tree distributions for each gene) to complete in as many days as there are genes (i.e., about a month for datasets with 32 genes, under two months for datasets with 50 genes). These analyses will still be computationally intensive, but not as intensive as waiting for all MrBayes analyses to converge. However, early terminations of MrBayes analyses are likely to make the analyses “invalid”, and potentially therefore reduce the accuracy of the resultant estimated species phylogeny. RAxML with bootstrapping can also be computationally intensive, so that analyses of single 100-taxon datasets with 1000 bootstrap replicates took on average almost 10 hours, making an analysis of 50 genes require 20 days just to obtain the initial set of tree distributions. Limiting RAxML to only 100 bootstrap replicates greatly reduces the running time to 1.5 hours on average per sequence alignment with 100 taxa, thus making it possible to obtain RAxML distributions on 50 genes in about 3 days.

The only computationally intensive method for estimating species trees from gene trees is BUCKy (all others complete extremely quickly). BUCKy’s computational challenges are very much impacted by the total number of taxa and total number of trees in its input. We compared BUCKy analyses based upon sparse samplings (at most 2000 trees per gene) from the MrBayes distributions as input to BUCKy, in comparison to larger numbers of trees (7000 to 15,000 per gene) on six 100-taxon datasets with 25 and 50 genes. This modification resulted a tremendous reduction in the computational effort, especially for the 50 gene datasets. The BUCKy analyses of these 50 gene datasets with this sparse sampling of MrBayes trees completed on average in about one day and had peak memory usage averaging 33 GB, while BUCKy analyses using the “full” MrBayes runs averaged one week per analysis and had average peak memory usage of 150 GB. Several of the BUCKy analyses of the full MrBayes sample used more than 200 GB of main memory, but no analysis of the sparse sample used more than 36 GB. The memory requirement for these full MrBayes analyses is truly prohibitive, since most investigators will not have computational resources of this scale available, but the sparse MrBayes analyses are computationally much less troublesome.

Except where indicated, the analyses in this study are based upon “fast” versions of MrBayes and RAxML with bootstrapping, as follows. MrBayes analyses were performed with only 1M iterations and we used only 100 bootstrap replicates for RAxML. Under these settings, single 100-taxon sequence alignments could be analyzed using MrBayes in under a day and using RAxML in about 1.5 hours. Therefore, analyses of 50 gene sequence alignments could be performed in 1-2 months using MrBayes and in about 3 days using RAxML. These settings made it possible to perform analyses of multiple datasets, each with tens of genes, in reasonable timeframes.

The fast methods we studied are all based upon FastTree, a method that is deterministic and very fast on even very large datasets. In our studies, even on the 500-taxon datasets with 50 genes, all the fast methods finished in under two hours and used less than 9 MB of peak memory.

**Figure 1:**
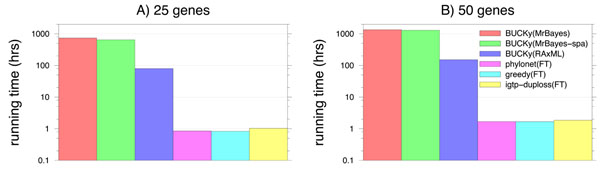
**Running time on 100-taxon non-ILS datasets** Average running time (y-axis given in log-scale) of methods on 100-taxon non-ILS datasets with MAFFT alignments of (A) 25 and (B) 50 genes. MrBayes performed two runs of 1M MCMC iterations in each analysis, with an average running time of 25 hours per sequence alignment. At the end of its analysis, MrBayes reported an average standard deviation of bipartitions at 0.065, indicating that it was far from convergence. BUCKy used 15K trees per gene for the full MrBayes distribution and 2K trees per gene on the sparse. RAxML performed 100 bootstrap replicates under GTRCAT. BUCKy analyses on RAxML used 100 trees per gene.

Figure 1 gives average running times of some representative methods on 100-taxon datasets with 25 or 50 genes, in which we ran MrBayes for only 1,000,000 iterations, and RAxML with only 100 bootstrap replicates. Even with these settings, however, methods based upon MrBayes are computationally intensive; BUCKy(MB-full) is the most expensive, using a month on 25 genes and close to two months on 50 genes. BUCKy(MB-spa) shaves about 4 days off that running time. The next slowest is BUCKy(RAxML) which finishes in under 4 days on 25 genes and about a week on 50 genes (note that these are analyses based upon 100 bootstraps, and that analyses using 1000 bootstraps instead of 100 would have been much slower). Finally, the methods based upon FastTree are extremely fast, finishing in under an hour on 25 genes and under two hours on 50.

### Evaluation of algorithm design choices

We begin the study with examining two specific algorithm design choices that were made in order to improve topological accuracy and/or running time.

Recall that Phylonet was designed to solve the MDC problem, and that when used with incompletely resolved gene trees, it is designed to solve the Constraint-MDC problem. The motivation for the Constraint-MDC problem is that low support edges are often not reflective of true history [20]. The hypothesis is that contracting the low support edges and solving Constraint-MDC will improve accuracy. Since FT-75% refers to the result of contracting all edges in the FastTree output with support below 75%, the hypothesis is that Phylonet(FT-75%) should be more accurate than Phylonet(FT). This is what we set out to test.

We also wished to evaluate the consequences of using only a sparse subset of the MrBayes distribution as input for BUCKy, instead of the full distribution. The advantage of using the sparse distribution is the decrease in running time and memory usage (as already noted); however, the concern is that using a sparse distribution might reduce accuracy of BUCKy analyses. We therefore wish to determine whether there is a statistical difference between BUCKy(MB-full) and BUCKy(MB-sparse).

In each experiment below, we began by comparing Phylonet(FT-75%) to Phylonet(FT) to see if there was a statistically significant difference. For those datasets in which we ran BUCKy(MB) analyses, we also compared BUCKy-con(MB-full) to BUCKy-con(MB-spa) and BUCKy-pop(MB-full) to BUCKy-pop(MB-spa). These pairwise comparisons were performed using Wilcoxon signed-ranks tests. Other comparisons between methods were performed using Wilcoxon signed-ranks tests, and then corrected for multiple tests using Bonferroni’s correction.

#### Phylonet(FT) vs. Phylonet(FT-75%)

We compared Phylonet(FT) to Phylonet(FT-75%) on every dataset we generated, and there was a statistically significant improvement obtained in every model condition (*p* < 0.01 for each condition, Wilcoxon signed-rank test). We observed a statistically significant improvement of Phylonet(FT-75%) over Phylonet(FT) on all the datasets without ILS datasets for both true and MAFFT alignments (*n* = 120 for each alignment type, *p* = 0.011 for the 500-taxon true alignments and and *p* < 0.0001 for all other datasets, Wilcoxon signed rank test). For the ILS datasets, we observed a statistically significant improvement for all 17-taxon ILS datasets (*n* = 500 for each number of genes and alignment type, *p* < 0.0001 Wilcoxon signed-rank test). On the ten 100-taxon ILS datasets, we also saw a statistically significant improvement of Phylonet(FT-75%) over Phylonet(FT) (*p* = 0.002 for the true alignments and *p* = 0.001 for the MAFFT alignments).

Since Phylonet(FT-75%) shows a statistically significant improvement over Phylonet(FT) in every model condition, it seems likely that contracting low support edges in FastTree trees does improve the species tree accuracy produced by Phylonet.

#### BUCKy(MB-spa) vs. BUCKy(MB-full)

We now evaluate the statistical significance of changes in tree error that result from using BUCKy to analyze a sparse subset of the MrBayes distribution instead of the full distribution. These comparisons were done on only a few datasets (25 17-taxon datasets with 32 genes, 25 17-taxon datasets with 8 genes, and six 100-taxon datasets with 25 genes). Of these three model conditions, there was only one condition (17 taxa with 32 genes) in which there was any statistically significant difference for BUCKy-con (*p* = 0.002, Wilcoxon signed-rank test), and no condition that resulted in a statistically significant change for BUCKy-pop.

This suggests that neither BUCKy-con nor BUCKy-pop is particularly impacted by using a sparse subset of the MrBayes distributions, and that the computational advantages of using the sparse distributions may be worth the risk involved in using fewer trees. However, it is also possible that the lack of statistical significance is a result of the small sample size, and that more extensive testing will demonstrate that the difference is statistically significant; thus, further research will need to investigate the impact of sparse sampling.

### Experiment 1: gene trees that can differ from the species tree due to ILS

We continue with the experiments we performed on 17-taxon and 100-taxon datasets where the true gene trees can differ from the true species trees due to ILS.

#### 17-taxon datasets

We begin the discussion with the results for 17-taxon datasets with 8 and 32 genes per dataset. These data evolved under Jukes-Cantor, the simplest substitution-only model (all substitutions are equally likely), and so there was no need to estimate the alignment on any dataset.

We begin with a discussion of Glass’s performance. First, Glass can be run on distance matrices estimated directly from an alignment or from a tree computed on an alignment. We evaluated the accuracy for trees returned by Glass on 25 datasets, each with 32 genes per dataset, using three different ways of calculating distances on 25 datasets: the logdet [35] distances, computed using DNADIST from the PHYLIP package (v3.69) [36], calculated directly from the alignment, and distances computed on the FastTree and MAP tree from the MrBayes analysis. The best results were obtained from the logdet distance matrix (66.6% FN error) with the other two distances having much higher error rates (76.9% for FastTree and 85.4% for the MAP tree). These results are all very poor, indicating that although Glass gives the best results on logdet distances, it is much worse than the other methods. Therefore, we omit Glass from any further discussion in this section.

**Figure 2:**
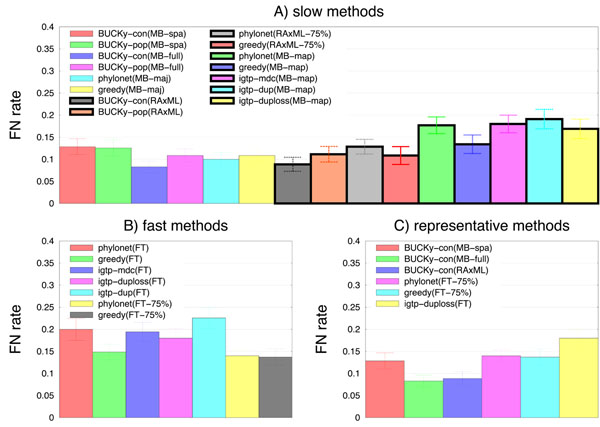
**Missing branch rates on 17-taxon 32-gene datasets with ILS** Average missing branch rates of methods on 25 17-taxon 32-gene datasets with incomplete lineage sorting. (A) shows results for the slow methods, (B) shows results for the fast methods, and (C) shows representative methods of both types. Bars indicate standard error.

Figure 2 shows results for all methods on 25 of the 17-taxon datasets with 32 genes, and we describe the differences between 8 and 32 genes. Although relative performance differs depending on the number of genes, some trends are consistent across the data. First, the topological accuracy is to a large extent predicted by the technique used to estimate the gene trees, with methods based upon MrBayes or RAxML distributions typically having less error than methods that use FastTree. For example, on 32 genes, methods based upon MrBayes (but not including the MB-map tree) or RAxML have error rates that range from 8.3-12.9%, while methods based upon FastTree have error rates that range from 13.7-22.6%. On 8 genes, methods based upon MrBayes range from 20-22.6%, methods based upon RAxML range from 19.4-24.9%, while methods based upon FastTree range from 21.7-32.0%. Thus, there is substantial overlap for results based upon MrBayes or RAxML (and even FastTree can produce fairly accurate trees for 8 genes), but clearly using better methods for gene tree estimation has an impact on the final species phylogeny. The best performing method differs for 8 and 32 genes (BUCKy-con(MB-full) is the most accurate for 32 genes, and BUCKy-con(RAxML) is the most accurate on 8 genes), but in general all BUCKy analyses do very well for both numbers of genes. Using a sparse subset of the MrBayes distribution reduces accuracy for BUCKy-con and BUCKy-pop under both numbers of genes, in some cases substantially (e.g., for 32 genes, BUCKy-con(MB-full) has 8.3% error but BUCKy-con(MB-spa) has 12.9% error).

Among the “fast” methods (i.e., the methods that use FastTree to estimate gene trees), Phylonet(FT-75%), Greedy(FT), and Greedy(FT-75%) are the best three for both numbers of genes, and iGTP-dup the least accurate for both numbers of genes. Finally, for both numbers of genes, the most accurate of the iGTP methods was iGTP-duploss.

We now compare six representative methods, BUCKy-con(MB-spa), BUCKy-con(MB-full), BUCKy-con(RAxML), Phylonet(FT-75%), Greedy(FT-75%), and iGTP-duploss(FT). The difference in error rate between the best fast method and the best slow method is about 5%, which in relative terms is large – but for datasets of this size, this is about 0.7 of an edge on average (since a 17-taxon tree has only 14 internal edges).

After using Bonferroni’s correction for multiple tests, we observed that only one pair of representative methods (iGTP-duploss(FT) and BUCKy-con(MB-spa)) had a statistically significant difference on 8 genes. For 32 genes, we saw some additionally statistically significant pairwise comparisons, all involving BUCKy-con(MB-spa) or BUCKy-con(MB-full). However, all the other methods could not be distinguished from each other. Furthermore, three fast methods–greedy(FT-75%), Phylonet(FT-75%), and iGTP-duploss(FT)–were statistically indistinguishable from BUCKy-con(MB-spa), BUCKy-pop(MB-spa), and BUCKy-pop(MB-full).

#### 100-taxon datasets

For the 100-taxon datasets, in order to run the computationally intensive methods, we explored performance on only 10 datasets, each with 25 genes. Because these datasets are large, we only evaluated BUCKy(MB-spa), terminating MrBayes at 1M iterations, and giving a sparse sample of 2000 trees for each gene to BUCKy. These datasets evolved under a model with substitutions as well as indels, and so we discuss results for both the true and MAFFT alignment on each dataset.

**Figure 3:**
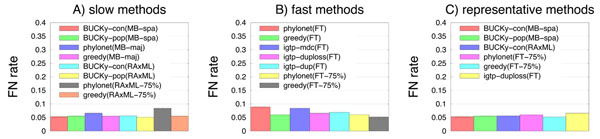
**Missing branch rates on true alignments of 100-taxon 25-gene datasets with ILS** Average missing branch rate of methods on ten (10) 100-taxon 25-gene datasets with incomplete lineage sorting on true alignments. (A) shows results for the slow methods, (B) shows results for the fast methods, and (C) shows results for representative methods of both types. Bars indicate standard error.

Glass, run on the logdet distance matrix computed directly from the sequence alignments, had very high error rates for both true and MAFFT alignments (73.4% and 74.2%, respectively), much higher than any other method (none of which had error rate above 9%). Error rates did not vary much between the MAFFT and true alignments, so that with the exception of Glass, error rates did not change by more than half a percent. Therefore, we focus our discuss on the results for the true alignment, and omit results for Glass. On the true alignments (see Fig. 3) all the methods had relatively low error rates that varied between 5.0% and 8.8%. Interestingly, there were several methods with error rates in the 5.0-5.2% range, each based upon a different technique for estimating the gene trees–BUCKy-pop(RAxML) with 5.0%, BUCKY-con(MB-spa) with 5.2%, and greedy(FT-75%) with 5.1% error. These observations suggest that these gene trees were relatively easy to estimate, in contrast to the 17-taxon datasets we studied earlier, where even the most accurate species trees based upon FastTree had distinctly higher error than the most accurate trees based upon either RAxML or MrBayes. Several fast methods have very good accuracy (errors in the 5.1%-5.9% range), including Greedy(FT), Greedy(FT-75%), and Phylonet(FT-75%), the three fast methods that had the least error for the 17-taxon datasets.

After correcting for multiple tests using Bonferroni’s correction, there were no statistically significant differences between the representative methods on the true alignments for these datasets, and only one statistically significant difference on the MAFFT alignment (between Phylonet(RAxML-75%) and BUCKy-pop(RAxML)).

### Experiment 2: analyses when no ILS occurs

Since a systematist will not always know the cause for incongruence between estimated gene trees, species tree estimation methods that are designed to handle ILS need to be able to reconstruct species trees when incongruence is due to other causes. Here we examine the case where all incongruence between estimated gene trees is due to estimation error.

**Figure 4:**
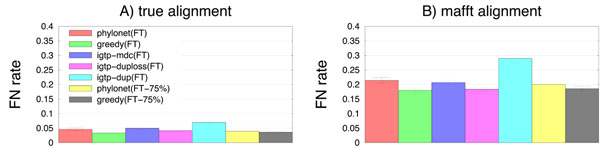
**Missing branch rates of fast methods on 100-taxon datasets without ILS** Average missing branch rate of fast methods on 120 100-taxon datasets without incomplete lineage sorting for 25 and 50 genes. (A) shows results for the true alignments and (B) shows results for the MAFFT alignments. Bars indicate standard error.

The true gene trees for these datasets have identical topologies but different branch lengths, thus allowing us to investigate the ability of methods to reconstruct the true species tree from gene tree estimates when no ILS occurs. These datasets evolved with indels as well as substitutions, thus requiring the estimation of alignments for the gene sequence datasets. When alignments are not highly accurate, however, gene trees will also have error, leading to the estimated gene trees exhibiting increased incongruence. We explored this question on datasets with 100 and 500 taxa. Trends for 500 taxa were very similar to those we observed for 100 taxa, and so we focus our discussion on the 100 taxon datasets. As before, Glass(logdet) had very high error, and we omit Glass from further discussion. The most striking observation is that the error rates of fast methods on the MAFFT alignment are much higher than error rates on the true alignment (Fig. 4) suggesting that alignment estimation error greatly impacted gene tree estimation error. The other general observation is that iGTP-dup(FT) has the highest error rates of all fast methods on both true and MAFFT. Therefore, for the rest of this discussion, we will only address the remaining methods.

All fast methods (besides iGTP-dup(FT)) have excellent accuracy on the true alignment, with error rates that vary from 3.4-4.9%; thus, the difference between the most accurate method (greedy(FT)) and the other fast methods (except for iGTP-dup(FT)) is very small. However, we note that the three fast methods with best accuracy are the usual ones – greedy(FT) with 3.4% error followed by greedy(FT-75%) with 3.6% error and Phylonet(FT-75%) with 4.0% error. After correcting for multiple tests, all but two comparisons are statistically significant; the only ones that aren’t are Greedy(FT) vs. Greedy(FT-75%) and iGTP-duploss(FT) vs. Phylonet(FT-75%).

The situation changes for the MAFFT alignments, where error rates for the fast methods are much higher (ranging from 18.0-21.4%, if we omit iGTP-dup(FT) which has 29% error), and the differences between methods somewhat greater. On these alignments, the most accurate method is Greedy(FT) at 18.0%, followed by iGTP-duploss at 18.4%, and then by Greedy(FT-75%) at 18.5%. Phylonet(FT-75%) is in fourth place with 20.0% error. Thus, the results for the MAFFT alignment are similar, but show greater differences between methods, and also show that iGTP-duploss(FT) can give very good results as well. After correcting for multiple tests, we find that the top three methods, Greedy(FT), Greedy(FT-75%), and iGTP-duploss(FT), cannot be distinguished statistically, nor can iGTP-mdc(FT) be distinguished from Phylonet(FT-75%) or Phylonet(FT).

**Figure 5:**
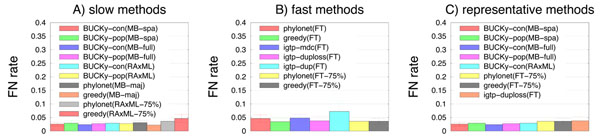
**Missing branch rates on true alignments for 100-taxon datasets without ILS** Average missing branch rate of methods on six (6) 100-taxon datasets without incomplete lineage sorting on true alignments for 25 and 50 genes. (A) shows results for the slow methods, (B) shows results for the fast methods, and (C) shows results for representative methods of both types. Bars indicate standard error.

In order to evaluate the computationally intensive methods, we used a subset of 12 datasets (half with true alignments and half with MAFFT alignments, and with half on 25 genes and half on 50 genes). Average error rates on the six datasets with the true alignment ranged from 2.2-3.0% when based upon MrBayes distributions (full or sparse), from 2.7-4.5% when based upon RAxML bootstrapping analyses, and from 3.3-7.0% when based upon FastTree (see Fig. 5). Thus, using MrBayes and RAxML instead of FastTree *did* improve the error, but not dramatically. The best accuracy for methods that use MrBayes was obtained by Greedy(MB-maj) (2.2% FN), followed closely by BUCKy-con(MB) at 2.5%. The best accuracy for methods based upon RAxML was BUCKy-pop(RAxML) at 2.7% FN, followed closely by BUCKy-con(RAxML) at 2.8%. The most accurate methods that use FastTree were Phylonet(FT-75%), Greedy(FT-75%), and Greedy(FT), with error rates of 3.5%, 3.5%, and 3.3%, respectively. iGTP-duploss(FT) had the lowest error of the three iGTP methods (3.7% FN rate), and iGTP-dup(FT) had the worst accuracy of all methods (7.0% FN rate). Not surprisingly, after correcting for multiple tests, none of the differences were statistically significant.

Results for the six datasets with the MAFFT alignment were much higher, but showed similar relative performance (data not shown). The best results for MrBayes analyses were obtained by BUCKy-con(MB-full) (14.0%), the best results for RAxML-based analyses were obtained by BUCKy-con(RAxML) (also at 14%), and the best results for FastTree-based analyses were obtained by Greedy(FT-75%) (at 14.8% FN rate). Here, too, iGTP-dup(FT) had the worst accuracy (29.5% FN rate) of all methods, and iGTP-duploss(FT) had the least error of all three iGTP methods (15.3% FN rate). Again, not surprisingly, after correcting for multiple tests, none of these differences were statistically significant.

### Overall summary of performance

The experiments we reported have datasets that range in terms of the causes for incongruence between estimated gene trees (some involve ILS, while others only involve estimation error), rates of evolution (the 17-taxon and 100-taxon non-ILS datasets have higher rates than the 100-taxon ILS datasets), presence of indels, number of taxa, and type of alignment (true or MAFFT). Thus, the relative performance between methods varies with the models. However, certain trends hold throughout.

The first observation is that BUCKy analyses of MrBayes or RAxML distributions produces highly accurate trees, either tied with the best or close to the best, even for the suboptimal way in which we ran these methods (stopping MrBayes analyses well before convergence, using only 100 bootstrap replicates for RAxML, and only using a sparse subset of the full MrBayes distributions).

The second observation is that several fast methods (notably, Greedy(FT-75%), Phylonet(FT-75%), and Greedy(FT)) provide very good results, coming close to BUCKy analyses of MrBayes and RAxML distributions (often without any statistically significant differences to these methods), and with greatly reduced computational requirements. Furthermore, these fast trees have very low computational requirements, completing in at most two hours and with peak memory usage of at most 9 MB, even on our largest datasets containing 50 genes and 500 taxa. Therefore, there are feasible alternatives to methods like BUCKy, which offer statistically-based estimation and high accuracy but at a computational cost that may be prohibitive.

The fast methods we studied are all based upon FastTree, a heuristic for ML that uses a deterministic hill-climbing heuristic to find locally optimal solutions to ML, and thus is likely to end up “stuck” in local optima. It is therefore possible that even better accuracy might be obtained, albeit at a computational cost. The remaining observations have to do with algorithm design. First, our study shows that methods, like Phylonet and BUCKy, that by design explicitly accommodate error in the input gene trees can produce more accurate trees than methods that assume all the input gene trees are correct. We have also shown that modifications to MrBayes (such as sparsification of its output distribution) can be made without compromising the accuracy of BUCKy’s estimated trees. These, and other approaches, could easily result in additional improvements for species tree estimation methods.

## Conclusions

Our study evaluating the computationally intensive methods was necessarily limited in number and in scope. Nevertheless, the experiments we performed show that some very fast methods for estimating species trees from gene sequence alignments can come close to the accuracy of the best methods, while taking dramatically less time (two hours instead of months of analysis), and with much smaller peak memory requirements (a few MB instead of potentially hundreds of GB). Furthermore, while we observed differences in accuracy between methods, some of which were substantial, the data suggests that in many cases, the differences between the best fast methods and the best computationally intensive methods we studied are not statistically significant. Additional experiments are clearly needed in order to evaluate whether this is true, or whether this is a consequence of the limited number of datasets we evaluated. It is also important to determine if these trends hold generally, or if there are conditions where the computationally intensive methods offer substantially improved accuracy.

Our study has many ramifications for simulation studies. Because alignment estimation error has an impact on both the absolute and relative performance of methods, future simulation studies should include indels in their models and use both estimated and true alignments. Gene tree estimation error has a very large impact on species tree estimation, and so the best methods for estimating gene trees should be used. This study shows that relative and absolute performance is also impacted by the number of taxa, and so datasets with larger numbers of taxa ought to be included. Finally, since simple methods (like the greedy consensus of estimated gene trees) are often quite accurate, these methods should be included in the methods that are compared.

Although we initiated the study in order to evaluate methods for estimating species trees in the presence of ILS, the observations in this study are also relevant to estimating species trees in the presence of gene duplication and loss. In particular, the poor performance of iGTP-dup for non-ILS datasets, in comparison to simple methods like Greedy, suggests that there is need for substantial improvement in methods for estimating species trees in the presence of gene duplication and loss. Perhaps the improvements for species tree estimation we observed by developing methods that explicitly handle error (either in a Bayesian framework, or by contracting low support edges in estimated gene trees) can be obtained for gene duplication and loss scenarios as well.

## Materials and methods

All datasets (true trees and true alignments) used in this study are available at http://www.cs.utexas.edu/users/phylo/datasets/ILS.

### Simulated datasets

#### 17-taxon datasets with ILS

The 17-taxon datasets with 8 and 32 genes are from [37], and were provided to us by Yun Yu and Luay Nakhleh. These datasets were generated by simulating Jukes-Cantor evolution (i.e., only with substitutions) down gene trees that evolved within species trees under a coalescent process, and each alignment contained DNA sequences of length 1000.

#### 100-taxon datasets with ILS

We simulated ten 100-taxon 25-gene datasets that evolve with ILS as follows. A single 100-taxon model tree from [28] was used as the species tree. We uniformly scaled down its branch lengths by 0.05 to produce a model tree with short enough branches so that ILS would occur. We evolved 25 trees within this species tree using MS [38], starting with an island model of 100 separate taxa, and then joining the lineages backwards in time based on the species tree. The MS command we used is:

ms 100 25 -T -I 100 1 1 ... 1 -ej <t> <from> <to> ...

This produces 25 gene trees, each with branch lengths. We simulated sequence evolution down each gene tree under a GTR+Gamma+Gap model (with parameters derived from the model tree in [28]) using ROSE, with the root sequence having length 1000. The GTR+Gamma+Gap parameters of model trees in [28] are based upon biological datasets, and the tree topologies and branch lengths are based upon a random birth-death model, and are generated using r8s [39].

#### 100- and 500-taxon datasets without ILS

We used model species trees from [28] (see discussion above). Each dataset contained 25 or 50 genes. Half of the datasets were generated by model trees that were identical to the species tree, and half were generated by model trees that were identical topologically but had different branch lengths. To produce the modified gene trees, each branch length in the species tree was multiplied by a different random number with expected value 1, and then rescaled so that all trees had the same total treelength. We then generated sequences on each model gene tree under GTR+Gamma+Gap using ROSE [25], using the model parameters given in [28], and with sequence length 1000 at the root.

## Competing Interests

The authors declare that they have no competing interests.

## Authors' contributions

TW conceived and designed the study; JY implemented the analysis tools, produced the data, and created the figures and tables; TW and JY analyzed the data; TW wrote the paper.
